# Development of a perioperative medicine research agenda: a cross sectional survey

**DOI:** 10.1186/1471-2482-4-11

**Published:** 2004-09-20

**Authors:** Nadia A Khan, Taha Taher, Finlay A McAlister, Andre Ferland, Norman R Campbell, William A Ghali, for the Canadian Perioperative Research Group

**Affiliations:** 1 Department of Medicine, University of Calgary, 3330 Hospital Drive NW, Calgary, AB, T2N 4N1, Canada; 2 Department of Medicine, University of Alberta, Walter Mackenzie Center, 8440-112 St., Edmonton, AB, T6G 2B7, Canada

## Abstract

**Background:**

Post-operative complications are a significant source of morbidity and mortality for patients undergoing surgery. However, there is little research in the emerging field of perioperative medicine beyond cardiac risk stratification. We sought to determine the research priorities for perioperative medicine using a cross sectional survey of Canadian and American general internists.

**Methods:**

Surveys were electronically sent to 312 general internists from the Canadian Society of Internal Medicine and 130 internists from the perioperative medicine research interest group within the US based Society of General Internal Medicine. The questionnaire contained thirty research questions and respondents were asked to rate the priority of these questions for future study.

**Results:**

The research topics with the highest ratings included: the need for tight control of diabetes mellitus postoperatively and the value of starting aspirin on patients at increased risk for postoperative cardiac events. Research questions evaluating the efficacy and safety of perioperative interventions had higher ratings than questions relating to the prediction of postoperative risk. Questions relating to the yield of preoperative diagnostic tests had the lowest ratings (p < 0.001 for differences across these categories).

**Conclusion:**

The results of this survey suggest that practicing general internists believe that interventions studies are a priority within perioperative medicine. These findings should help prioritize research in this emerging field.

## Background

Over 40 million people undergo non-cardiac surgery in the United States each year [1]. Postoperative cardiac complications affect 2–18% of patients alone, costing over 20 billion dollars annually in the United States [2]. Many will suffer other potentially avoidable perioperative complications such as pneumonia, hemorrhage or infection. Efforts to minimize these complications have resulted in the development of the field of perioperative medicine.

Until now, research in this emerging field has focused primarily on cardiac risk stratification [3]. Despite the significant medical and economic burden of perioperative complications, few studies evaluate diagnostic testing, risk stratification for non-cardiac complications, or interventions to prevent cardiac or non-cardiac complications. Thus, there is a growing need to expand the research base in this field.

Given the wide spectrum of comorbidities in the surgical patient and the potential for postoperative complications, numerous research questions still need to be answered. The purpose of this study is to identify top perceived research priorities in this field using a survey of general internists practicing perioperative medicine.

## Methods

### Participants

To obtain the opinions of general internists who practice perioperative medicine, from the Canada and the United States, all general internists within the Canadian Society of Internal Medicine (n = 312) and all members of the perioperative medicine interest group of the American based Society of General Internal Medicine (n = 130), were surveyed. Physicians were excluded if they practiced in a subspecialty rather than general medicine (>90% of perioperative medicine consults are performed by general internists [4]) or did not perform preoperative consultation.

### Survey development and administration

Research questions for the survey were generated from a Medline search of perioperative medicine studies and from a focus group of five general internists active in perioperative medicine research. The questionnaire was pre-tested by four independent general internists for clarity and to confirm face validity. Modifications were made based on this pre-testing. The questionnaire was developed in English and then translated into French for French speaking Canadian physicians by a medical translator. Subsequently, a bilingual general internist validated the French translation.

The questionnaire contained 30 randomly ordered research questions conceptually divided into three themes: 1. evaluating the yield of preoperative diagnostic tests (5 questions), 2. predicting postoperative risk (6 questions) and 3. determining the efficacy of perioperative interventions (19 questions). A subset of the sample (130) was asked to specifically rank the importance of these three categories as an internal check of the reliability of the survey instrument. This subset was also asked to list any other research questions that ought to be considered for future research apart from those included in the questionnaire.

Respondents were asked to rate the priority for each research question to be studied in the future on a 10 point Likert scale where 1 indicates a low priority study question and 10 is a high priority research question. For questions that have been partially answered by existing studies, respondents were specifically asked to rate the priority of "further research" in these areas.

The self- administered questionnaire was electronically mailed in October 2000 (faxes were sent to those without e-mail addresses). For non-responders, second and third mail-outs were sent in November and December 2000.

### Statistical analyses

Descriptive statistics were used to analyze the demographic data and ratings for individual questions. T-tests (two- sided) were used to compare mean response scores across different subgroups. Because of the multiple planned comparisons, the alpha was set at 0.005 to determine statistical significance. Repeated measures analysis of variance was used to analyze differences across the 3 major themes of questions using SPSS statistical software.

## Results

After 3 mailings, we obtained 152 completed surveys (overall response rate 34%). Thirty-three respondents were then excluded because of subspecialty status or because they did not perform perioperative consults.

Respondents belonging to the Society of General Internal Medicine special interest group and Canadian Society of Internal Medicine were identical in most respects except fewer Canadian Society of Internal Medicine members had academic appointments (54% vs. 100%). Table 1 shows the demographic and professional characteristics for all respondents.

**Table 1:** Physician characteristics (n = 119)

Characteristics	
Age (SD)	44 (9.2)
Female (%)	24
Average year of graduation	1982
Number of years in practice, mean (range)	13 (0.5–35)
Practice Location (%)	
Rural	8
Urban <50 000	8
Urban 50–250 000	25
Urban >250 000	59
Academic appointment (%)	77
Number of preoperative consults performed per month, median (range)	10 (1–100)

Research questions evaluating the efficacy of perioperative interventions had higher ratings followed by questions relating to prediction of postoperative risk. Questions evaluating the yield of preoperative diagnostic tests had the lowest ratings. The differences in ratings across these general categories were statistically significant (p < 0.001) and this pattern persisted regardless of academic status or volume of consults seen.

Mean scores for individual research questions, based on responses where 1 indicates low priority for future research and 10 indicates high priority, ranged from a low of 3.6 (± 2.3 standard deviation) to a high of 7.2 (± 2.1 standard deviation). Mean scores for the ten highest rated individual questions are given in Table 2. Only one respondent suggested additional research topics that were not included in the questionnaire. The full list of 30 research topics presented to respondents in the questionnaire is presented in the appendix [see Additional file 1].

**Table 2:** The ten highest rated perioperative research issues: mean scores* (rank)

Research issues	Total	95% (CI)	High** volume	Low volume	Academic	Non-academic
The value of tight control of diabetes mellitus postoperatively.	7.2 (1)	6.8–7.6	7.2 (1)	7.1 (2)	7.1 (3)	7.2 (1)
Starting aspirin on patients at increased risk for postoperative cardiac complications.	7.1 (2)	6.7–7.5	7.2 (2)	7.0 (4)	7.1 (4)	7.1 (2)
Safety and efficacy of continuing aspirin preoperatively for those already taking aspirin.	7.0 (3)	6.6–7.4	7.0 (5)	7.1 (1)	7.1 (5)	7.0 (3)
Optimal management of perioperative anticoagulation for patients with prosthetic valves.	7.0 (4)	6.5–7.4	6.9 (6)	7.1 (3)	7.0 (6)	7.0 (4)
The value of starting angiotensin converting enzyme inhibitors for those at increased risk of postoperative cardiac complications.	6.9 (5)	6.5–7.3	7.2 (3)	6.7 (9)	7.0 (7)	6.9 (7)
Determining the diagnostic yield of routine postoperative cardiac surveillance.	6.8 (6)	6.4–7.2	7.1 (4)	6.6 (13)	6.5 (12)	6.9 (6)
Developing interventions to minimize postoperative delirium.	6.8 (7)	6.4–7.2	6.5 (9)	7.0 (6)	6.4 (14)	6.9 (5)
The value of starting beta-blockers for patients at increased risk of postoperative cardiac complications.	6.7 (8)	6.2–7.2	6.4 (11)	7.0 (5)	7.2 (1)	6.6 (10)
Optimal management of perioperative anticoagulation for patients with atrial fibrillation.	6.6 (9)	6.2–7.1	6.3 (12)	6.9 (7)	6.6 (11)	6.7 (9)
Developing a risk stratification index for predicting postoperative pulmonary complications	6.6 (10)	6.2–7.1	6.6 (8)	6.7 (10)	6.5 (13)	6.7 (8)

* Mean scores were derived from responses based on a 10-point Likert scale. A score of 10 indicates high priority and 1 indicates low priority for future research.

** Ratings from consultants who see a high volume of preoperative consults (>10/month).

Mean scores for most questions were similar among physicians independent of their academic status or whether they performed a high volume of preoperative consults (defined as greater than 10 consults per month) or not. For several questions, however, differences in ranking according to academic status and volume of consultations were found, although none achieved statistical significance (Table 2).

## Discussion

There are few areas within perioperative medicine that are well studied beyond the area of predicting cardiac risk. The American Heart Association perioperative guidelines highlighted the paucity of studies on interventions to prevent postoperative cardiac events [3]. Reflecting this statement, respondents globally rated studies that determine the efficacy and safety of interventions as higher priority for future research compared to studies predicting postoperative risk or determining diagnostic yield of tests. Within the category of intervention studies, questions on medical therapy to prevent postoperative cardiac complications were among the highest rated questions. This result is congruent to the significant prevalence, morbidity and mortality associated with postoperative cardiac complications. A study by Devereaux et al also identified this area as an important target for future research after finding considerable practice variation in the management of cardiac medications [4]. Innovative interventions for cardiac protection with antiplatelet agents, angiotensin converting enzyme inhibitors or tight glycemic control were highly rated. Despite the publication of small trials of beta blocker therapy, responding internists felt that there was a need to definitively determine the efficacy of perioperative beta blockade. Respondents may be more skeptical of adopting results from the small trials of beta blocker where methodological controversies have arisen [5]. Other intervention questions that were rated highly focused on understudied areas of perioperative anticoagulation. Determining optimal perioperative anticoagulation strategies for patients with prosthetic valves or atrial fibrillation was rated highly.

Although perioperative risk stratification is well studied for identifying those at risk of cardiac events, pulmonary complications occur more frequent than cardiac complications and are associated with a longer hospital stay [6]. Reflecting this significant morbidity and cost of respiratory complications, development of a prediction rule for postoperative pulmonary complications was among the highest rated topics.

Since the completion of the survey, the ratings also reflect ongoing research activity within perioperative medicine. Tight glycemic control was a high rated topic and a study examining the effect of tight glycemic control in postoperative patients in a critical care setting has been published since the completion of this survey. Also since the completion of this survey, a large multi-center trial examining the efficacy of perioperative beta blockade has been launched. Another highly rated research topic was developing a prediction rule for pulmonary complications. Recently, a study was published to identify those at increased risk of postoperative pneumonia. Two of the highly rated research priority topics have been recently published in major medical journals [7,8].

There are several potential limitations with this study. The low response rate may be felt to limit the generalizability to other general internists. However, views of those who practice perioperative medicine and who have a particular interest in the area, the research consumers, are important in developing a research agenda. We presume that physicians who practice within an area and who have a particular interest are more likely to respond to a survey than those who do not. Thus, the physicians who responded to the questionnaire and reported practicing perioperative medicine define the group of research consumers we were targeting. This study also only examined the beliefs of general internists on research priorities since greater than 90% of the perioperative medicine consultations are conducted by general internists rather than subspecialists in internal medicine in tertiary care centers [4]. Also, lower response rates are seen in physician surveys but surveys with response rates of 10–45% are published in major medical journals [9-15]. Another limitation is that anesthesiologists, cardiologists or primary care physicians perform perioperative medicine consultations and their opinions were not elicited in this survey. Another potential limitation is that not all important research questions could be examined. However, only one respondent indicated additional research topics suggesting that there were no major omissions in the research topics listed. Additionally, it is difficult to interpret meaningful differences in mean Likert response scores among individual questions. Individual scores and their confidence intervals should be interpreted to give a general idea of high- priority questions, rather than a strict ranking of research pursuits.

## Conclusion

Perioperative medicine research is growing in response to the significant medical and economic consequences of perioperative complications. Identifying the research priorities of those who provide perioperative medical care – the consumers of research, is important. The results suggest that intervention studies are a higher priority for future research compared to studies that predict postoperative complications or determine the yield of diagnostic tests. Researchers and funding boards may use the findings of this survey to identify perceived high priority research topics within perioperative medicine.

## Competing interests

None declared.

## Authors' contribution

NK, TT, AF, NC, FAM, and WG contributed to the design of the project. NK and TT contributed to data collection and data analysis. NK, TT, WG contributed to writing the manuscript. AF, NC, FAM made substantive, intellectual editing contributions.

## Pre-publication history

The pre-publication history for this paper can be accessed here:



## Supplementary Material

Additional File 1 Perioperative medicine research questions. This appendix lists all 30 perioperative research issues from the survey, along with the mean scores rated by all respondents. Click here for file
