# Supplementary material for: Development of a perioperative medicine research agenda: a cross sectional survey
Source: BMC Surg. 2004 Sep 20;4:11. doi: 10.1186/1471-2482-4-11 (PMC521487; doi:10.1186/1471-2482-4-11)
Supplement: Additional File 1 — Perioperative medicine research questions. This appendix lists all 30 perioperative research issues from the survey, along with the mean scores rated by all respondents. [file 1471-2482-4-11-S1.doc]

**Appendix. Perioperative medicine research questions (mean score)**

Studies that evaluate efficacy of perioperative interventions**:**

1. Should we hold or continue thiazide type diuretics in the preoperative period? (4.12)
2. Should we hold or continue loop diuretics in the preoperative period? (3.59)
3. Should we hold or continue anti-platelet agents (e.g. ASA) preoperatively for nonvascular surgery? (7.04)
4. Should we hold or continue non-steroidal anti-inflammatory drugs preoperatively? (5.19)
5. Should we start aspirin on patients who are at increased risk for post- operative cardiac events (i.e. those with coronary artery disease or risk factors for coronary artery disease)? (7.13)
6. Should we start beta - blockers on patients who are at increased risk for post operative cardiac events (i.e. those with coronary artery disease or risk factors for coronary artery disease)? (6.74)
7. Should we start nitrates on patients who are at increased risk for post- operative cardiac events (i.e. those with coronary artery disease or risk factors for coronary artery disease)? (6.37)
8. Do pro-kinetic agents given preoperatively to patients with gastro-esophageal reflux disease reduce the risk of aspiration pneumonia? (4.97)
9. Should we start perioperative bronchodilators for patients who have symptoms or signs of COPD but who have not been on bronchodilators previously? (6.06)
10. What is the optimal management of perioperative anticoagulation for patients with prosthetic heart valves? (6.97)
11. What is the optimal management of perioperative anticoagulation for patients with atrial fibrillation? (6.65)
12. How tightly should diabetes mellitus be controlled postoperatively? (7.18)
13. How tightly should diabetes mellitus be controlled before surgery? (6.55)
14. Is a restrictive strategy of red cell transfusion equivalent to a liberal strategy in stable patients who have undergone non-cardiac surgery? (5.92)
15. Should we use prophylactic beta - blocker therapy for those patients at high risk for postoperative atrial fibrillation? (6.37)
16. Development of interventions to prevent postoperative delirium. (6.78)
17. If there was a contraindication to beta blocker therapy, should we start dihydropyridine calcium channel blockers on patients who are at increased risk for postoperative cardiac events (i.e. those with coronary artery disease or risk factors for coronary artery disease)? (5.98)
18. Should we start ACE inhibitors on patients who are at increased risk for postoperative cardiac events (i.e. those with coronary artery disease or risk factors for coronary artery disease)? (6.90)
19. If there was a contraindication to beta blocker therapy, should we start non-dihydropyridine calcium channel blockers on patients who are at increased risk for postoperative cardiac events (i.e. those with coronary artery disease or risk factors for coronary artery disease)? (6.21)

# Studies which develop postoperative risk stratification schemes:

1. What is the risk of postoperative stroke for patients with asymptomatic carotid stenosis of varying degrees? (6.18)
2. What is the risk of postoperative seizure for patients with known seizure disorder? (4.72)
3. Development of new indices to risk stratify patients for postoperative cardiac complications. (5.02)
4. Development of new indices to risk stratify patients for postoperative pulmonary complications (e.g. aspiration pneumonia, respiratory failure). (6.65)
5. Development of an index to stratify patients at risk for postoperative delirium. (5.74)
6. What are the perioperative complications for patients with varying degrees of hepatic dysfunction? (5.34)

# Studies which evaluate yield of perioperative diagnostic tests:

1. What is the diagnostic yield of routine postoperative cardiac surveillance (i.e. cardiac enzymes + ECG) in patients who are at high risk for cardiac events? (6.81)

1. What is the diagnostic yield of routine preoperative blood work (i.e. complete blood count, electrolytes, creatinine, and coagulation screen)? (4.78)
2. What is the diagnostic yield of routine preoperative chest x-ray? (4.62)
3. What is the diagnostic yield of preoperative spirometry? (5.33)
4. What is the diagnostic yield of routine preoperative anti-epileptic drug levels for patients with known seizure disorder? (4.57)
